# Event-Related Potentials Discriminate Familiar and Unusual Goal Outcomes in 5-month-Olds and Adults

**DOI:** 10.1037/dev0000376

**Published:** 2017-08-14

**Authors:** Christine Michel, Katharina Kaduk, Áine Ní Choisdealbha, Vincent M. Reid

**Affiliations:** 1 Department of Psychology, Heidelberg University, and Max Planck Institute for Human Cognitive and Brain Sciences, Leipzig, Germany; 2 Department of Psychology, Lancaster University; 3 Department of Psychology, Lancaster University, and Economic and Social Research Institute, Dublin, Ireland; 4 Department of Psychology, Lancaster University

**Keywords:** action perception, event-related potentials, semantic processing, N400, PSW

## Abstract

Previous event-related potential (ERP) work has indicated that the neural processing of action sequences develops with age. Although adults and 9-month-olds use a semantic processing system, perceiving actions activates attentional processes in 7-month-olds. However, presenting a sequence of action context, action execution and action conclusion could challenge infants’ developing working memory capacities. A shortened stimulus presentation of a highly familiar action, presenting only the action conclusion of an eating action, may therefore enable semantic processing in even younger infants. The present study examined neural correlates of the processing of expected and unexpected action conclusions in adults and infants at 5 months of age. We analyzed ERP components reflecting semantic processing (N400), attentional processes (negative central in infants; P1, N2 in adults) and the infant positive slow wave (PSW), a marker of familiarity. In infants, the PSW was enhanced on left frontal channels in response to unexpected as compared to the expected outcomes. We did not find differences between conditions in ERP waves reflecting semantic processing or overt attentional mechanisms. In adults, in addition to differences in attentional processes on the P1 and the N2, an N400 occurred only in response to the unexpected action outcome, suggesting semantic processing taking place even without a complete action sequence being present. Results indicate that infants are already sensitive to differences in action outcomes, although the underlying mechanism which is based on familiarity is relatively rudimentary when contrasted with adults. This finding points toward different cognitive mechanisms being involved in action processing during development.

The ability to detect, process and interpret human actions is perhaps one of the most complex components of social cognition. It is therefore remarkable that the capacity to engage with observed actions and identify actions as goal directed in nature is present in infancy (see Gredebäck & Daum, 2015; Ní Choisdealbha & Reid, 2014 for an overview). A critical but mainly unaddressed issue remains; namely which processes, such as attentional or semantic processes, underlie action understanding at different ages. The current study aims to shed light on the neural processes taking place during action perception in early infancy and in adulthood. We examined neural correlates of the processing of expected and unexpected action conclusions in the context of food consumption, one of the first observed and experienced crucial actions in infancy.

Infants are remarkably good at understanding other people’s movements as goal directed actions (Gredebäck & Daum, 2015). Infants’ action understanding has mainly been studied using behavioral measures such as looking times, pupil dilation or anticipatory looking. Infants start to anticipate the goal of a grasping action between 6 and 12 months (Falck-Ytter, Gredeback, & von Hofsten, 2006; Kanakogi & Itakura, 2011) and this ability is related to their own grasping skills (Kanakogi & Itakura, 2011). Similar results were found for food consumption. At 6 months at the latest, infants anticipate that a cup or a spoon will be brought to the mouth (Hunnius & Bekkering, 2010; Kochukhova & Gredebäck, 2010). Not only do infants at 6 months of age have expectations about the end state of an action they observe, they are also able to evaluate whether an expected consequence occurred or not. This process has mostly been assessed with measures that reflect violation of expectation. In the context of grasping, infants as young as 6 months of age show longer looking times if an action consequence does not match with their expectations raised by the physical appearance of a grasp (Daum, Vuori, Prinz, & Aschersleben, 2009) or with their expectation about other people’s goals (Woodward, 1998). With regard to feeding actions, starting at 4 months of age, infants seem to be more surprised when the bowl of a spoon is placed on the back of another person’s hand (unexpected action outcome) than in the person’s mouth (expected action outcome), as indicated by differences in pupil dilation (Gredebäck & Melinder, 2010, 2011). Thus, infants very early in life possess the ability to anticipate and evaluate other people’s goal directed actions. The above-mentioned studies used behavioral measures to investigate infants’ action understanding. These studies leave the question open on *how* infants detect and interpret goal directed actions. This limitation can be overcome using neurophysiological measures like Event-Related Potentials (ERPs). ERPs have a high temporal resolution and consist of well-defined components reflecting different steps during stimulus processing including semantic processing, allocation of attention, or memory updating. Critically, these processes may be active to a different degree at differing points in development (Reid et al., 2009).

With regard to action evaluation, the N400 event-related potential component has been related to semantic mismatch within adult populations when a perceived action violated expectations in a current context (see Amoruso et al., 2013 for an overview of the N400 in action contexts). An enhanced N400 was found in response to movie sequences of actions that included unexpected action outcomes in the context of eating (e.g., empty spoon put to mouth) as compared to expected outcomes (e.g., spoon conveying food put to mouth; Reid & Striano, 2008). Another study presented images depicting the crucial stages of an action in sequence (Reid et al., 2009). Expectations about the action outcome were raised by 2 images of an ongoing action (Image 1 context: e.g., a person holding a pretzel; Image 2 action execution: a person bringing the pretzel to the mouth) while a third image presented either an expected action conclusion (the pretzel in the mouth of the person) or an unexpected action conclusion (the pretzel at the ear of the person). In adults, an N400 component was elicited only in response to the unexpected outcome, reflecting a mismatch in the semantic processing of this action. The same effect was found in 9-month-olds indicating that infants at this age anticipate the outcome of an expected or unexpected action via the use of semantic processing systems. However, no N400 effect was found with infants at 7 months of age, although the negative central (Nc) component, related to attention mechanisms (Reynolds, 2015; Reynolds & Richards, 2005), differentiated conditions (Reid et al., 2009).

One explanation for this finding is that younger infants do not utilize semantic systems during action processing. Rather, discrimination between conditions is due to mechanisms related to attention, which according to Reid et al. (2009) was reflected in differences in the Nc component. As the Nc component is associated with allocation of attention (Reynolds, 2015; Reynolds & Richards, 2005) and is enhanced for familiar when compared to unfamiliar stimuli in infants (de Haan & Nelson, 1999), the highly familiar and evolutionarily significant event of eating elicited more activation on this component (Reid et al., 2009). An alternative explanation is that younger infants found the paradigm, comprising a sequence of three images, to be too complex for optimal processing. The presented three-step sequence of context, action execution and action conclusion may challenge infants’ working memory capacity specifically at the younger age group (Ross-Sheehy, Oakes, & Luck, 2003). This overload in information may inhibit semantic processing. It may therefore be possible that even infants younger than 9 months of age possess the ability to process action information in a semantic manner, but the rather complex paradigm may have been unsuitable to elicit evidence for this ability. To address this alternative explanation, the present study reduced the complexity of the stimulus presentation: instead of presenting the complete three-step sequence of context, execution and conclusion (as in Kaduk et al., 2016; Reid et al., 2009), we presented only the picture of the action conclusion to the infants. We assume that this simplified presentation facilitates the processing of the stimuli, as no other information (i.e., action context, action execution) need to be kept in mind to evaluate the end state of the action. This assumption is in line with studies showing that reducing the complexity of stimuli influences the neurophysiological processes taking place in ERP studies (Peykarjou, Pauen, & Hoehl, 2014, 2016; Ross-Sheehy et al., 2003). From a practical standpoint, it was anticipated that these single-image stimuli would be more likely to be tolerated by young infants than multiple-image sequences, resulting in better data quality and more trials for inclusion in ERP averages. To investigate neural correlates of early action understanding, we tested 5-month-olds. As behavioral results show, infants around this age are able to anticipate and evaluate eating actions (Gredebäck & Melinder, 2010, 2011; Hunnius & Bekkering, 2010; Kochukhova & Gredebäck, 2010), we therefore chose to examine 5-month-olds as we were particularly interested in the early neural correlates of action understanding, asking the question - is semantic processing already functioning when infants have just started to understand other people’s actions, or do other processes, like attention, develop before semantic processing? Given that neural correlates of action perception have not been widely studied in a 5-month-old cohort, hypotheses for the infant sample included multiple possible neural correlates of action perception. If a less complex presentation of the action conclusion enables even younger infants to process the stimuli semantically, we hypothesized that an N400 would be found in response to the unexpected action conclusion. On the other hand, a lack of action understanding or the missing context may lead to no differences or to differences on a more basic processing level. This could be reflected in an enhanced Nc component for the expected condition indicating allocation of attention to the salient eating action (Reynolds, 2015; Reynolds & Richards, 2005) as it was the case in 7-, and 9-month-olds (Reid et al., 2009). Another plausible component to differentiate between conditions is the PSW. Even though it has not previously been investigated in the context of action understanding in infants, it is related to memory updating processes of only partially encoded stimuli (Nelson, 1997; Riby & Doherty, 2009; Snyder, 2010; Snyder, Garza, Zolot, & Kresse, 2010; Webb, Long, & Nelson, 2005). An enhanced PSW for the unexpected condition would reflect the increased neural resources which are needed to encode this action outcome. This would conversely show that the expected action outcome is already more familiar to the infants. Differences on the PSW would inform us about infants’ familiarity with the action outcomes. Any differences in these ERP components in response to the expected and unexpected action outcome stimuli could indicate whether the associated processes (N400: semantic processing, Nc: allocation of attention, PSW: familiarity) are functional during action processing at 5 months of age. Considering the results of the current study in addition to the prior literature related to the Nc and the N400 in 7- and 9-month-old infants (Reid et al., 2009) will provide us with informative insights into cognitive mechanisms taking place during action perception in the first postnatal year of life.

To further investigate the role of the context of an action, we also tested an adult sample with the same paradigm. As we kept the stimuli and the timing of the action conclusion pictures identical to Reid et al. (2009), comparing our results to the adult results in Reid et al. (2009) allowed us to directly examine the influence of the presented action context and action execution on the neural processing of expected and unexpected action conclusions. For the adult sample, we hypothesized the following - in line with Reid et al. (2009), we expected to find an N400 component in response to only the unexpected action conclusion in the adult sample (see also Mudrik, Lamy, & Deouell, 2010). As we presented photographic images of actions as stimuli, a frontocentral distribution of the N400 was expected (Amoruso et al., 2013; Ganis, Kutas, & Sereno, 1996). In the study by Reid et al. (2009), attentional mechanisms were involved in the processing of the stimuli in 7-, and 9-month-old infants as reflected in an Nc component of greater magnitude for the expected condition. This enhanced allocation of attention possibly indicated the high salience and evolutionary significance of the depicted eating action. In the adult sample, we therefore analyzed differences between conditions on the P1 component (Vogel & Luck, 2000) which is associated with arousal and the N2 component, which is associated with processes of orientation of attention and is suggested to be a successor to the infant Nc component (Folstein & Van Petten, 2008; Rothenberger, Banaschewski, Siniatchkin, & Heinrich, 2007).

## Method

### Participants

All participants were recruited following a local media campaign for volunteers, from the area in and around Stockton-on-Tees, North East England. This study was conducted with the understanding and the written consent of each participant’s caregiver or the participant in accordance with institutional protocols.

#### Infants

The final analysis was comprised of the data of 15 5-month-old infants (average age: 152 days, range = 147–167 days; 11 male, 4 female). The sample size is within the normal range for infant ERP studies (Stets, Stahl, & Reid, 2012) and is comparable to the sample size of the 7-month-olds (*n* = 13) and the 9-month-olds (*n* = 14) in the study by Reid et al. (2009) that we have based our study on. The sex of the infant participants was not equally distributed, but as we did not have any expectations about how the sex of the participants would influence the results, we have no reason to believe that this unequal distribution impacts the validity of our study. Another 7 infants (2 female, 4 male, 1 unknown) were tested but had to be excluded from the final sample because they failed to reach the minimum 10 artifact-free trials per condition (*n* = 5), or because of technical failure (*n* = 2). All infants had to be born full term (37–42 weeks gestation). No other exclusionary criteria were applied. Infants were given a t-shirt and £10 (approximately 13$) was given to the parents to cover travel costs.

#### Adults

The adult sample consisted of 27 adults who were undergraduate students with normal or corrected to normal vision. All tested adults were included in the final analyses. Adult participants received £7 (approximately 9$) to participate.

### Stimuli

**Figure 1 fig1:**
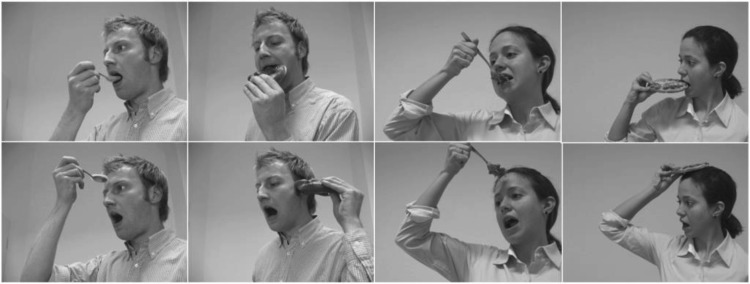
Stimulus material depicting eating. Top line displays the expected action; bottom line displays the unexpected action both for the male and female actor. Images were displayed in color to participants. All photographed individuals in this article provided written consent permitting their images to be published.

The stimuli were photographs depicting a male or a female actor, showing eating actions in two different ways: either with a spoon or holding the food. Those actions were presented either in an *expected* manner (food in mouth) or in an *unexpected* way (food touching other parts of the head). Figure 1 shows all stimulus pictures that were used in the study. Each participant saw each of the eight different stimuli. Stimuli were presented at full screen size (26 cm × 34 cm) on a 60-Hz 17-inch height adjustable stimulus monitor at a viewing distance of 90 cm. This produced a visual angle of 16.44° × 21.39°.[Fig-anchor fig1]

### Procedure

During recording, infants sat on their caregiver’s lap in a dimly lit 2 × 2 m testing area which was separated from the rest of the laboratory by black colored room dividers. A camera located above the center of the presenting screen recorded infants’ looking behavior. If an infant became fussy or uninterested in the stimuli, the experimenter gave the infant a short break and attempted to resume the study when the infant was once again alert and calm. The testing session ended when the infant’s attention could no longer be attracted to the screen. EEG was recorded continuously during the presentation.

The experiment consisted of a block of 32 action conclusion photographs with a division of male–female stimuli and expected-unexpected trials of exactly half each. The block could be repeated 9 times resulting in a maximum of 288 stimulus presentations. The two conditions were presented to the participant in a pseudorandomized order with the constraint that the same condition was not presented more than three times consecutively. Stimuli were presented utilizing the *Stim*^*2*^*-Gentask* computer software package by Neuroscan Compumedics (Charlotte, U.S.A.).

**Figure 2 fig2:**
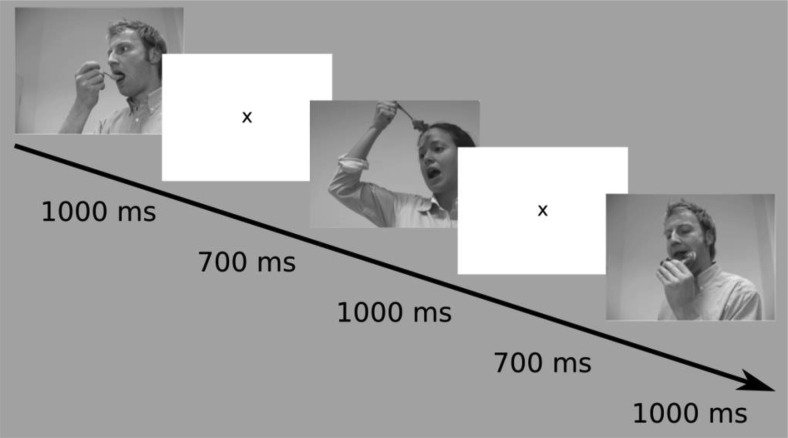
An example of the stimuli sequence presented to the participants: From top left to bottom right: expected-spoon (1,000 ms), interstimulus interval (700 ms), unexpected-spoon (1,000 ms), interstimulus interval (700 ms), expected-holding food. All photographed individuals in this article provided written consent permitting their images to be published.

Each ERP time-locked image was presented on the screen for 1,000 ms. Between the presentation of each image, the screen was white for a period of 700 ms, only displaying a fixation cross in the center of the screen (see Figure 2 for an example of the stimulus presentation sequence). A 1,700-ms period in between the onset of one critical stimulus and the next was used based on previous work with infants by Friedrich and Friederici (2011).[Fig-anchor fig2]

### EEG Recording and Analysis

EEG was recorded continuously from 32 scalp locations according to the 10–20 system, referenced online to AFz using Ag-AgCl ring electrodes with a sampling rate of 1 Khz. For infants, the quality of the ongoing EEG data was inspected visually, and individual electrodes were examined if required, with the application of more paste should an electrode be too noisy or displaying channel offsets. For the adult sample, impedances were kept lower than 10kΩ. Horizontal and vertical electro-oculograms (HEOG + and VEOG+) were recorded bipolarly and the EEG data was amplified via a Neuroscan 32-channel amplifier. For additional data editing, the software EEGLAB version 13.4.4b was used (Delorme & Makeig, 2004). Raw data were filtered offline with a 0.3 to 30-Hz bandpass filter using the pop_eegfiltnew function in EEGLAB and rereferenced offline to the averaged mastoids (TP9, TP10). Data were segmented into epochs of waveform that comprised 200 ms prior to stimulus onset and 1,000 ms following stimulus onset. Baseline was corrected using the 200 ms before stimulus onset. Following review of the video recordings of infant behavior, all trials in which the infant did not pay attention to the stimuli for the full 1,000 ms of stimulus presentation were rejected from further analysis. On average, this included 53 trials in the expected (range = 24–99 trials) and 50 (range = 20–101 trials) in the unexpected condition in the infant sample. No significant difference between the amount of trials rejected based on the video analysis in the expected and in the unexpected condition were found, *t*(14) = 1.49, *p* = .159. The majority of trials were rejected because infants did not attend to the trials at all (mean of 37 trials in the expected and mean of 35 trials in the unexpected condition). In contrast, it was only in the minority of the excluded trials that infants attended to the trials at some point but not during the whole 1000s (mean of 16 trials in the expected and mean of 15 trials in the unexpected condition). For both measures (amount of trials infants did not attend to the screen at all and amount of trials infants only paid attention to the stimulus during onset), we did not find differences between both conditions, *t*(14) = 1.49, *p* = .159 and *t*(14) = 0.54, *p* = .596, respectively. All remaining trials were scanned for artifacts using the automatic artifact detection implemented in ERPLAB version 5.0.0.0 (Lopez-Calderon & Luck, 2014). A trial was excluded from further analysis whenever the peak-to-peak amplitude in any channel exceeded a threshold of 200 μV in a 200-ms window. Window steps were set to 100 ms (Wahl, Michel, Pauen, & Hoehl, 2013). The remaining segments were visually and manually edited for artifacts and blinks. Finally, data were averaged for the expected and the unexpected condition.

On average each infant contributed a mean of 31 trials (*SD* = 12.95, range = 15–54) to their average for the expected conclusion of the action condition and a mean of 32 trials (*SD* = 14.48, range = 3–66) for the unexpected conclusion of the action condition.

For the adult sample, analyses relied on a mean of 99 trials in the unexpected (*SD* = 25.99, 25–135) and 99 in the unexpected condition (*SD* = 25.09, range = 28–136) with a minimum of 25 and 28 included trials, respectively.

## Results

The level of significance was set to 0.05 if not stated otherwise and Greenhouse-Geisser correction was applied if applicable. Grand average of all channels for the infant sample can be found in supplementary material 1.

### Infants

#### N400

**Figure 3 fig3:**
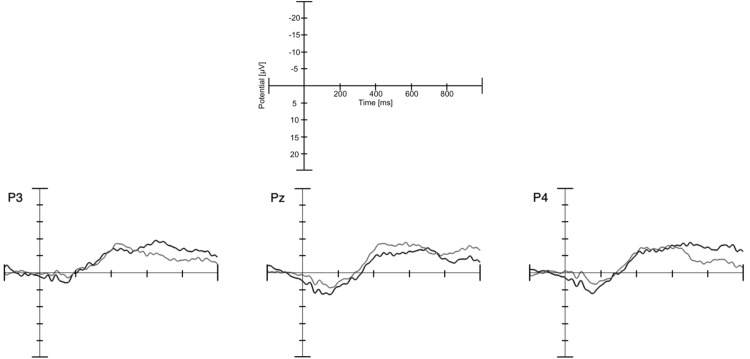
Channels analyzed for the N400 component in the infant sample. Black lines show the expected and gray lines refer to the unexpected condition. Note that negative is plotted up.

Although an N400 analysis might have been pursued on the basis of previous work (Reid et al., 2009) and to establish whether the simplified stimuli would elicit such an effect in a younger age group, visual inspection did not show any evidence for an N400 (see Figure 3). In the 9-month-olds in Reid et al. (2009), the N400 component was present in the unexpected condition and absent in the expected condition. To detect such differences in the morphology between ERP waves, for example the presence of a component in one condition and the absence of a component in the other condition, an analysis, as described by Hoormann, Falkenstein, Schwarzenau, and Hohnsbein (1998), can be performed. To conduct this analysis, the values of the amplitude of the ERP wave are extracted at several time points for both conditions and compared in a repeated measures analysis of variance (ANOVA) with within-subject factors of time and condition. If ERP waves differ in their morphology, the interaction between the factors time and condition will reach significance. To test for an N400 effect in our sample, we conducted the same analysis as with the infant participants in Reid et al.’s (2009) action observation study. However, we included only 15 instead of 17 time windows to be able to appropriately estimate the parameters given our sample size. Using the same time window (612 to 780 ms) and the same electrodes (P3, Pz, P4), a 2 × 15 repeated measures ANOVA with the within-subjects factors condition (expected vs. unexpected) and time (15 samples at one per 12 ms) was performed. As the signal of some participants may cross the *x*-axis in the selected time window, data were normalized for each participant and each condition using the following quotient to calculate the values for each time point

$$value\;at\;each\;time\;point=\frac{mean\;amplitude}{normalization}$$

[Fig-anchor fig3] with

meanamplitude=meanvalueofP3,PzandP4forthistimepoint

$$normalization=\frac{\sum \;normalized\;mean\;amplitudes\;of\;all\;time\;points}{number\;of\;time\;points}$$

$$\begin{array}{l} normalized\;mean\;amplitude\\ =\;mean\;amplitude\;of\;each\;time\;point\\ -\;minimal\;amplitude\;of\;\mathrm{all}\;time\;points\;of\;this\;participant\;and\;condition \end{array}$$

A significant Time × Window interaction would indicate a difference in morphology. No Condition × Time interaction was found, *F*(3.00, 42.01) = 1.47, *p* = .236.

Infants’ initial expectations about the presented eating action may have been overwritten by repeatedly seeing a person holding food to the head in the course of the experiment. To test for this idea, we performed the same analysis only for the first half of valid trials for infants that contributed more than 20 trials to each condition. This analysis included 11 infants. No significant Condition × Time interaction was found, *p* = .547.

#### Nc

The mean amplitude for the Nc was assessed in left frontocentral (FP1, F3, FC5 and C3), frontocentral (Fz and Cz) and right frontocentral (FP2, F4, FC6 and C4) electrode clusters in a time window between 350 and 600 ms after stimulus onset, which fitted the resultant morphology and was congruent with other studies investigating this waveform (Hoehl, Reid, Mooney, & Striano, 2008; Kaduk et al., 2016). A 2 × 3 repeated measures ANOVA was conducted with the within-subjects factors condition (expected vs. unexpected) and region of interest (left vs. central vs. right). This analysis revealed only a significant interaction between condition and region of interest, *F*(1.39, 19.50) = 5.27, *p* = .024, η_p_^2^ = 0.273, all other *p*s > 0.321. As post hoc repeated measures ANOVAs confirmed, this interaction was due to differences in the amplitude between the regions of interest only in the expected condition (*F*(2, 28) = 6.50, *p* = .005, η_p_^2^ = 0.317). No such difference was found for the unexpected condition, *p* = .879. Level of significance for post hoc ANOVAs was set to *p* < .025. Follow-up paired *t* tests revealed that amplitude over the left hemisphere in the expected condition was more negative than over the right hemisphere, *t*(14) = −3.671, *p* = .003. When comparing the expected and unexpected conditions separately for each region of interest with paired *t* tests, no significant difference was found, all *p*s > 0.061. Level of significance for the post hoc paired *t* tests was set to *p* < .017 for Bonferroni correction.

#### PSW

**Figure 4 fig4:**
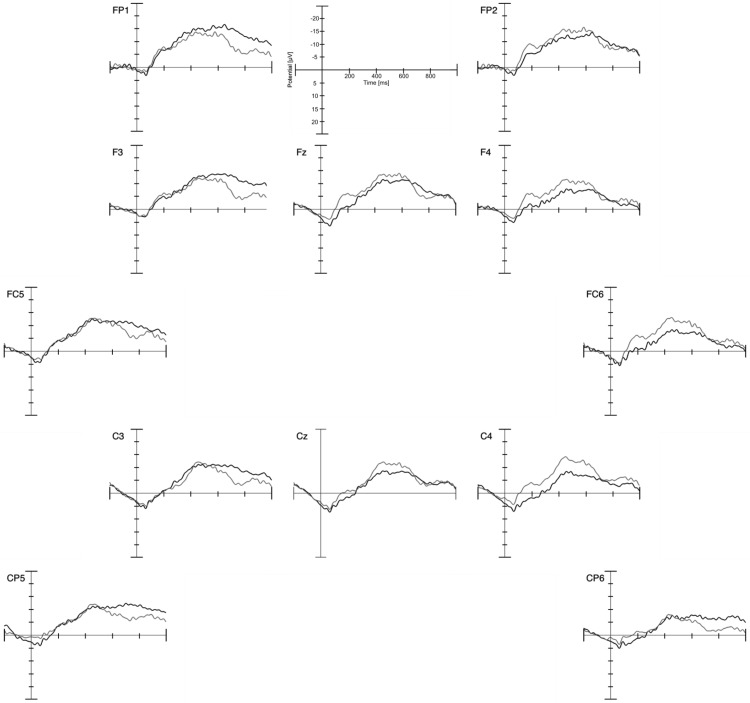
Channels analyzed for the negative central (Nc) and positive slow wave (PSW) in the infant sample. Black lines show the expected and gray lines refer to the unexpected condition. Note that negative is plotted up.

The 650- through 900-ms time window for the PSW analysis was selected due to the morphology of the data. Although this time window is shorter and earlier than the PSW window typically used in other studies (de Haan & Nelson, 1997; Webb et al., 2005), visual inspection of the data (see Figure 4) showed the slow wave tapering off before 1,000 ms poststimulus. Data were analyzed accordingly and in accordance with procedures used in other studies reporting earlier PSW effects (Reid, Striano, Kaufman, & Johnson, 2004; Striano, Kopp, Grossmann, & Reid, 2006) and hemisphere specific differences (Csibra, Tucker, & Johnson, 2001; Parise, Friederici, & Striano, 2010; Parise, Reid, Stets, & Striano, 2008; Reid et al., 2004). A 2 × 2 repeated measures ANOVA with within subject factors condition (expected vs. unexpected) and hemisphere (right vs. left) was conducted with the mean amplitude on left (FP1, F3, FC5, C3, CP5) and right (FP2, F4, FC6, C4, CP6) frontocentral channels in a time-window of 650–900 ms. Channels were chosen with regard to visual inspection of the grand averages and the existing literature showing that the PSW is most prominent on frontotemporal electrodes (de Haan & Nelson, 1999; Reid et al., 2004; Snyder, Webb, & Nelson, 2002).[Fig-anchor fig4]

Results revealed no significant main effect of condition, *p* = .134, however a significant main effect of hemisphere was found, *F*(1, 14) = 8.10, *p* = .013, η_p_^2^ = 0.367. The interaction between hemisphere and condition showed a significant effect, *F*(1, 14) = 6.13, *p* = .027, η_p_^2^ = 0.305. Level of significance for post hoc tests comparing both conditions separately for the left and the right hemisphere was set to *p* < .025 for Bonferroni correction. Paired sample *t* tests revealed that conditions differed significantly from each other only over the left hemisphere *t*(14) = −2.56, *p* = .023, *d* = 0.660, not over the right hemisphere, *t*(14) = −0.211, *p* = .836, *d* = 0.055. Over the left hemisphere, mean amplitude was more positive for the unexpected condition (*M* = −6.36, *SE* = 2.05) as compared to the expected condition (*M* = −10.92, *SE* = 2.20). No such difference was found over the right hemisphere (*M* = −5.29, *SE* = 2.34 for the unexpected condition and *M* = −5.66, *SE* = 2.34 for the expected).

### Adults

The level of significance was set to 0.05 if not stated otherwise and Greenhouse-Geisser correction was applied if applicable. Grand average of all channels for the adult sample can be found in supplementary material 2.

#### N400

**Figure 5 fig5:**
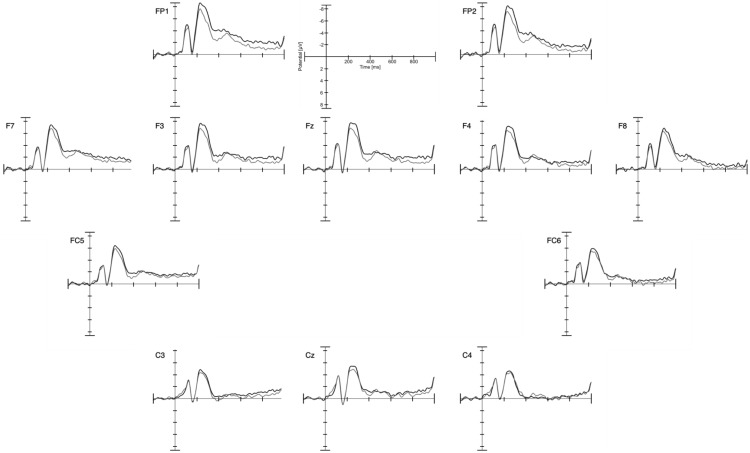
Channels analyzed for the N400 and the N2 component in the adult sample. Black lines show the expected and gray lines refer to the unexpected condition. Note that negative is plotted up.

As in Reid et al. (2009) the N400 component was only visible in the unexpected condition, whereas no N400 was visible in the expected condition. To test EEG data for differences in morphology between conditions, Hoormann et al. (1998) suggest a window analysis. Therefore we exported in total 13 amplitude values every 12 ms between 400–544 ms over frontocentral channels (FP1, FP2, F3, Fz, F4, F7, F8, FC5, FC6, C3, Cz, C4) where the N400 was most prominent. Again, as the signal of some participants may cross the *x*-axis in the selected time window, data were normalized for each participant and each condition using the same normalization quotient as for the infant data. A repeated measures ANOVA with the within-subject factors condition (expected vs. unexpected) and time (13 time points) was conducted. A significant Condition × Time interaction would suggest that the ERP waves differ between conditions, for example, that the N400 would be present in only one condition. The ANOVA revealed a significant Condition × Time interaction, *F*(3.84, 99.93) = 3.06, *p* = .022, η_p_^2^ = 0.105. This significant interaction between condition and time highlights that there are differences in the morphology between the ERP waves of the two conditions. As can be seen in Figure 5, the N400 was only present in the unexpected condition but not in the expected. No main effects were found, all *p*s > 0.069.[Fig-anchor fig5]

#### P1

**Figure 6 fig6:**
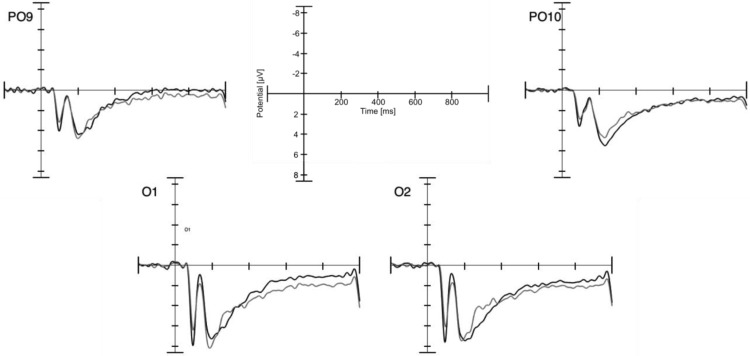
Channels analyzed for the P1 component in the adult sample. Black lines show the expected and gray lines refer to the unexpected condition. Note that negative is plotted up.

The visual component P1 is known to appear 80–130 ms after stimulus onset on occipital areas (Hillyard & Anllo-Vento, 1998). To investigate effects on the P1, mean amplitudes on left (O1 and PO9) and right (O2 and PO10) occipital channels in the time-window 80–130 ms served as the dependent variable (see Figure 6). A 2 × 2 repeated measures ANOVA with the within-subject factors condition (expected vs. unexpected) and hemisphere (left vs. right) only yielded a significant main effect of condition, *F*(1, 26) = 5.83, *p* = .023, η_p_^2^ = 0.183, with a more positive amplitude for the expected condition (*M* = 3.71, *SE* = 0.44) than for the unexpected condition (*M* = 3.27, *SE* = 0.40). No other main effect or interaction was found, all *p*s > 0.428.[Fig-anchor fig6]

#### N2

The N2 component was analyzed on left frontocentral (FP1, F7, F3, FC5 and C3), frontocentral (Fz and Cz) and right frontocentral (FP2, F8, F4, FC6, C4) electrode clusters in the time-window 200–350 ms (Folstein & Van Petten, 2008). A 2 × 3 repeated measures ANOVA with the within-subject factors condition (expected vs. unexpected) and region of interest (left vs. central vs. right) only yielded a significant main effect of condition, *F*(1, 26) = 9.71, *p* = .004, η_p_^2^ = 0.272, with a more negative mean amplitude for the expected (*M* = −5.15, *SE* = 0.41) than for the unexpected condition (*M* = −4.44, *SE* = 0.35). All other *p*s > 0.292.

## Discussion

In this study, we examined the neural correlates that were associated with the perception of expected or unexpected action conclusions in early infancy and adulthood. In infants, the present experiment found that the PSW, but not the N400 or the Nc, differentiated expected and unexpected action outcomes at 5 months of age. The PSW was enhanced for the unexpected condition relative to the expected condition on left frontal channels. As the PSW is related to memory updating processes for stimuli that are only partially encoded (Nelson, 1997; Riby & Doherty, 2009; Snyder, 2010; Snyder et al., 2010; Webb et al., 2005), the result suggests that enhanced activity was required to process the unexpected, thus unfamiliar action conclusions when contrasted with processing the expected, more familiar ones. Infants are sensitive to differences in action outcomes in early development. But the mechanisms by which this is displayed indicate that the cognitive systems employed are relatively rudimentary, as they are based on familiarity and memory encoding processes. In adults, an enhanced N400 component occurred only in response to the unexpected action outcome, suggesting semantic processing of this action type even without the context of an action sequence being present. Results on the P1 and the N2 components indicate that attentional processes are active in adulthood similar to 7- and 9-month-old infants (Reid et al., 2009), at least when observing actions that are related to food consumption.

In our infant sample, no N400 component was produced for the unexpected condition when contrasted with the expected condition, even when we analyzed only the first half of trials to check for potential learning effects during the course of the experiment. There is currently some evidence that infants at 9 months of age use semantic systems to process actions (Kaduk et al., 2016; Reid et al., 2009), although no such studies have been conducted with infants as young as those investigated in the current study. In Reid et al. (2009), the complexity of the stimuli may have been one potential cause for a lack of N400 effect found in infants at 7 months of age. The present study attempted to simplify the stimuli yet aimed to still contain violations of expectation related to action outcomes in one condition but not the other. Despite simplification of the stimuli to facilitate processing, no N400 component was found. One explanation of this finding is, that 5-month-old infants do not utilize semantic systems when observing others’ action outcomes. Another possible explanation for the lack of an N400 effect is that infants need an action context and need to perceive how an action is executed to semantically process that action. To test this idea, one could test 5-month-olds with the three-step action sequence presentation present in Reid et al. (2009). Given that even 7-month-olds did not show signs of semantic processing in that paradigm, we would not expect N400 effects to occur. Another possibility for future research would be to examine 7- and 9-month-olds with our simplified paradigm. This way, the influence of the complexity of the stimulus presentation could be tested against the influence of embedding an action outcome into an action sequence.

Despite the lack of an N400 effect, the ERP waveform showed other components of interest in relation to infant processing of actions. The Nc component was observed in the morphology of the ERP waveform in both conditions. The mean amplitude of the Nc in both conditions differed significantly from baseline with *t*(14) = −3.652, *p* = .003 for the expected condition and *t*(14) = −6.164, *p* < .001 for the unexpected condition. However, there was no statistical difference in the mean amplitudes of the Nc between conditions. This is in contrast to the results found in 7- and 9-month-olds that showed an enhanced Nc component in response to the expected condition that was related to eating (Reid et al., 2009) and consequently in contrast to our hypothesis. One possible explanation for this lack of difference in the Nc component may be that the mere presence of food itself elicits allocation of attention in 5-month-olds, whereas 7-month-olds are already more sensitive to the action of actually *eating* food instead of the mere presence of food. As the Nc was equally distinct in both conditions, we cannot conclude that attentional mechanisms play no role in action understanding in young infants. However, our results show that attentional mechanisms did not discriminate between expected and unexpected goal outcomes.

In the present work, the mean amplitude of the PSW differed between conditions over frontal channels of the left hemisphere. The fact that the PSW differed between conditions only over the left hemisphere aligns with studies that have previously reported left frontal ERP effects in infancy from 4 to 6 months of age (Csibra et al., 2001; Parise et al., 2008, 2010). The PSW has been related to familiarity detection, as it decreases with increased exposure to a stimulus (Snyder, 2010; Snyder et al., 2010) and when updating a memory representation of a partially encoded stimulus (Nelson, 1997; Webb et al., 2005). In the current study, the PSW was enhanced in response to the unexpected as compared to the expected condition. Thus, more activity was needed to encode the unexpected action outcome than the expected action outcome. This suggests that the unexpected action conclusion was most likely perceived as more novel and unfamiliar to the infants, whereas the expected outcome was already familiar and therefore elicited less prominent slow wave activity. The result on the PSW analysis suggests that infants at 5 months of age process actions at the level of familiarity versus novelty. It is therefore possible that differences in the PSW only occurred because infants were perceptually more familiar with food in the mouth than food at the head. It follows that this unfamiliarity elicited the enhanced PSW in the unexpected condition without awareness of what defines the novelty of this stimulus, that is, that the displayed action is unusual.

The findings of the present study help to refine our knowledge of action understanding in early development and suggest that other processes precede semantic processing of action. These processes, as shown in the present study and in previous work (Reid et al., 2009), are likely to involve detection of familiarity and, later in development, allocation of attention to the presented stimuli. Further work is required to understand the earliest emergence of the semantic processing system and how its application to action processing corresponds to its application in language processing (Kaduk et al., 2016).

It is assumed that the reduction in complexity of the stimuli in the present study when contrasted with those used in Reid et al. (2009) will help to facilitate infant processing of the difference between expected and unexpected actions. This has not been verified via any independent means, such as assessing overall looking time or gaze shift patterns. Combining neurophysiological and behavioral measures would allow us to depict the broader picture of processes taking place during action understanding. A simultaneous application of both measurements very often seems impractical as different measures have different requirements (e.g., different timing of stimuli for different measures, required number of trials). Nonetheless, using the same stimuli in paradigms with different methods may be a promising next step for future research (Hoehl, Wahl, & Pauen, 2014; Wahl et al., 2013). For instance, an increase in pupil dilation in response to the action outcomes presented with and without the action context would inform us about the role of the presented action context for infants action understanding (Gredebäck & Melinder, 2010). Such combined methods are currently under development and, despite added complexities, stand to yield a number of advances in infancy research (Domínguez-Martínez, Parise, Strandvall, & Reid, 2015; Wass, de Barbaro, & Clackson, 2015).

In the present study, food stimuli were used because 5-month-old infants are familiar with feeding actions and observe their caregivers performing those actions multiple times daily. It is currently an open question whether other familiar but less motivationally salient object-directed actions, such as the phone- and hairbrush-related actions used in Hunnius and Bekkering (2010), elicit similar or distinct patterns of neural activity in infants of this age group. If the PSW effect in the present study was mainly driven by perceptual familiarity with the action, we would expect similar results to other actions which infants are familiar with.

As we kept the stimuli and the timing of the action conclusion picture identical to the study by Reid et al. (2009), adult results of both studies can be directly compared. In our adult sample, a N400 occurred only in response to the unexpected action outcome, reflecting the processing of a semantic mismatch for the unfamiliar action condition. This result is in line with studies that found an enhanced N400 in response to unfamiliar or unexpected action outcomes using video stimuli (Reid & Striano, 2008; Sitnikova, Holcomb, Kiyonaga, & Kuperberg, 2008) or pictures (Mudrik et al., 2010). It replicates the results of Reid et al. (2009) and therefore suggests that no action context is needed for adults to process actions in a semantic way.

In addition to the effects on the N400, enhanced P1 and N2 amplitudes were found in response to the expected condition. As stimuli were controlled for luminance, we do not consider that these differences are due to psychophysical characteristics. However, an increased P1 is associated with higher arousal (Vogel & Luck, 2000). The N2 is associated with an orientation of visual attention in oddball paradigms (Folstein & Van Petten, 2008). The fact that both components are enhanced for the expected condition (related to eating) is in line with the infant results in Reid et al. (2009) showing an enhanced Nc component, indicating more allocation of attention, to the expected action. In accordance with the interpretation of Reid et al. (2009), an eating action is a highly salient event and of high evolutionary significance that may therefore lead to more arousal and attention than the unexpected condition. Interestingly, the similarities in the function and the assumed neural source of the N2 and the Nc led to the suggestion that the Nc may be a precursor in infants to the adult N2 (Rothenberger et al., 2007). This may explain the analogous results - the enhanced activity for the expected condition - in our adult sample and the infant sample in Reid et al. (2009). However, see Marinović, Hoehl, and Pauen (2014) for a study that did not find corresponding results for infants and adults on the N2 in an oddball paradigm.

As the paradigm in our study and the one used in Reid et al. (2009) differ in the substantial aspect of generating a complete context of an action including the execution of the context itself, direct comparisons of both studies are not valid except with the adult participants. However, when taking the differences in the paradigms into account, the results from the current study, when combined with the results by Reid et al. (2009), give us insight into the neural mechanisms underlying action perception in the first postnatal year of life and in adulthood. When presented with only an action conclusion, the infant brain at 5 months of age detects differences between expected and unexpected action outcomes. This is likely due to familiarity, as shown by differences in the PSW. At 7 months, action understanding is indexed via differences in attentional mechanisms, as evidenced by changes in the Nc (Reid et al., 2009) in the context of an action sequence. Finally, at 9 months of age, in addition to the enhanced attention to the salient eating stimulus, the N400 is present when a complete action sequence is presented. This indicates that semantic processing is involved in the processing of actions in a way that it continues into adulthood. For adults, even the presentation of the final action conclusion is sufficient for a semantic system to be activated in the detection of an unfamiliar action. The utilization of ERPs enabled us to disentangle the different underlying processes that drive action understanding at different points during development. Testing different age groups with the same paradigm in future studies, for example testing 7- and 9-month-olds with our simplified stimuli, will help to disentangle the influence of the complexity of the presentation and the influence of the action context.

In conclusion, the results of this study demonstrate that infants at 5 months of age are capable of discriminating expected and unexpected actions, and that this is manifested at the level of neural activity. The finding that PSW was involved in this dissociation between conditions rather than other components which index higher levels of processing, such as attention or semantics, suggest that at 5 months of age infants utilize a relatively simple mechanism for detecting such differences based on familiarity. How this capacity relates to more complex forms of action processing, such as grasping the concept of affordance for tools as seen in later infancy, is yet to be understood. Adults however use a semantic system to make sense of actions even when an action sequence is missing.

## Supplementary Material

10.1037/dev0000376.supp
