# Supplementary material for: Event-Related Potentials Discriminate Familiar and Unusual Goal Outcomes in 5-month-Olds and Adults
Source: Dev Psychol. 2017 Aug 14;53(10):1833–43. doi: 10.1037/dev0000376 (PMC5611762; doi:10.1037/dev0000376)
Supplement: Supplementary file 2 [file z2p999173932so2.docx]

**Supplemental Materials**

**Event-Related Potentials Discriminate Familiar and Unusual Goal Outcomes in 5-Month-Olds and Adults**

**by C. Michel et al., 2017, *Developmental Psychology***

**http://dx.doi.org/10.1037/dev0000376**


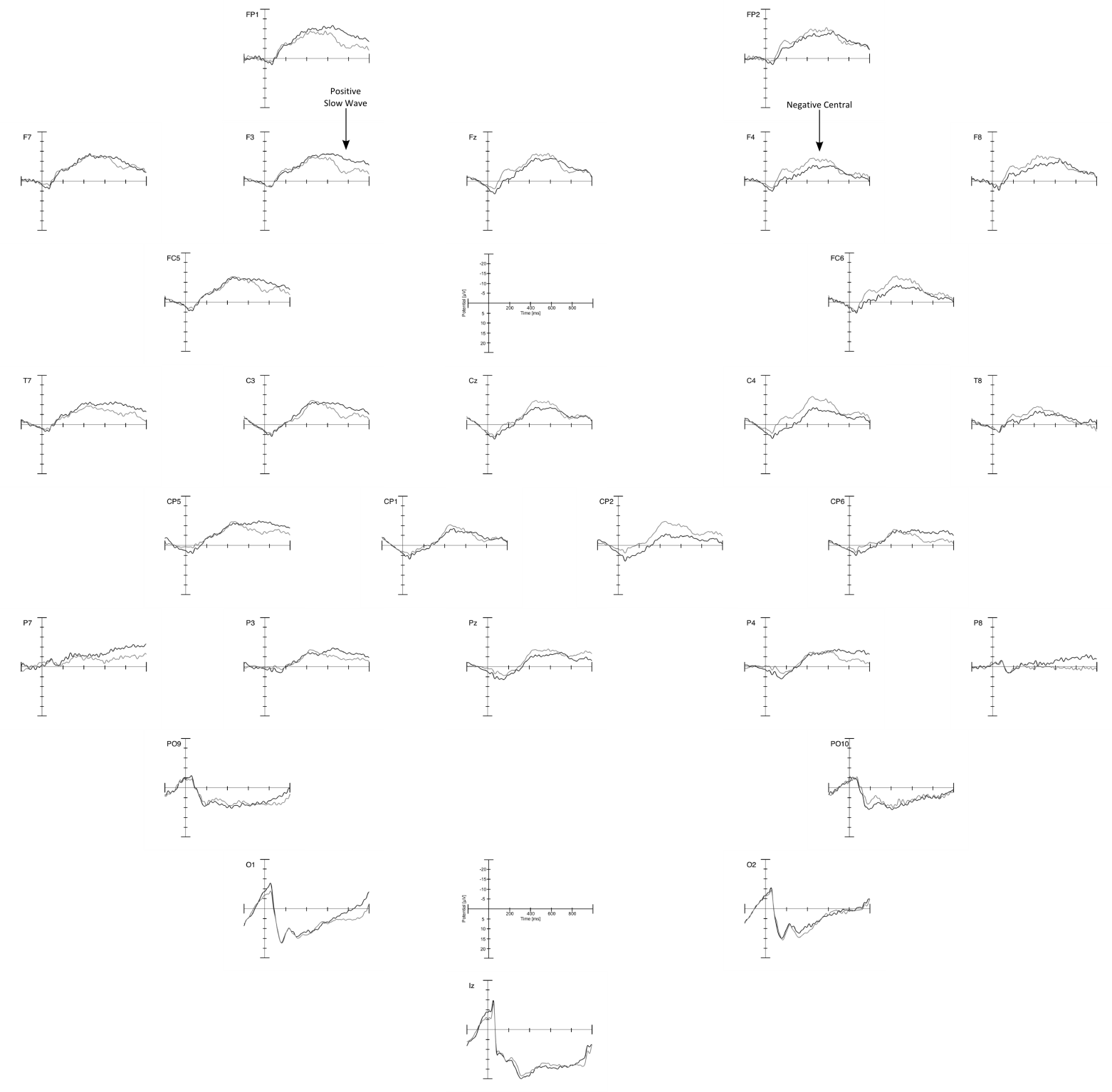
Supp. 1: Grand average of all channels for the infant participants. Black lines show the expected and grey lines refer to the unexpected condition. Note that negative is plotted up.
